# Equity in the allocation of oncology bed resources in China: Longitudinal data from 2008 to 2022

**DOI:** 10.1371/journal.pone.0356697

**Published:** 2026-08-19

**Authors:** Xingyue Pu, Zhigang Zhong

**Affiliations:** 1 Department of Organization and Personnel, Sichuan Provincial Maternity and Child Health Care Hospital, Sichuan Provincial Women’s and Children’s Hospital, The Affiliated Women’s and children’s Hospital of Chengdu Medical College, Chengdu, Sichuan Province, China; 2 Office of Sichuan Cancer Prevention and Control Center (Department of Prevention), Sichuan Clinical Research Center for Cancer, Sichuan Cancer Hospital & Institute, Sichuan Cancer Center, Affiliated Cancer Hospital of University of Electronic Science and Technology of China, Chengdu, Sichuan Province, China; National Center for Chronic and Noncommunicable Disease Control and Prevention, Chinese Center for Disease Control and Prevention, CHINA

## Abstract

**Objective:**

Cancer has emerged as one of the most significant public health challenges in the 21st century. With rapidly growing demand for oncology services, the allocation of oncology bed resources is closely associated with the accessibility and effectiveness of cancer diagnosis and treatment. This study presents a comprehensive assessment of equity in oncology bed allocation across Chinese hospitals, providing evidence to inform policy decisions for optimizing cancer care resource distribution.

**Methods:**

Data were extracted from the China Health Statistics Yearbook (2007–2023), and analyzed using Lorenz curves, Gini coefficients(G), and Theil index (T) to evaluate the equity of oncology bed resource allocation in China.

**Results:**

From 2008 to 2022, the number of oncology beds in Chinese hospitals showed an increasing trend, but the growth rate slowed down. According to Lorenz curves, the equity of oncology bed allocation by population was better than by geographic area. Compared to 2008, the population-based equity improved in 2022, while the geographic area-based equity showed no improvement. Based on G, the population-based allocation of oncology beds from 2008 to 2022 had G between 0.15 and 0.20, indicating absolute equity with an overall declining trend, while the geographic area-based allocation maintained G around 0.69, consistently showing severe inequity. According to T, population-based allocation demonstrated better equity with T all below 0.1, whereas geographic area-based allocation showed poorer equity with indices all above 0.6. For both population and geographic dimensions, within-group T were greater than between-group indices, indicating that inequity primarily stemmed from intra-regional disparities.

**Conclusion:**

While China’s oncology bed resources are expanding in scale, they are facing dual challenges of imbalanced “population-geography” allocation and significant regional differences. We should focus on improving geographical accessibility and regional balance to help alleviate the ‘difficulty in hospitalization’ for cancer patients.

## Introduction

Cancer has emerged as a critical public health challenge in the 21st century, with deaths caused by cancer accounting for nearly one-sixth of global deaths and three-tenths of premature mortality (ages 30–69) [1], imposing a substantial disease burden. In 2022, there were 18.74 million new cancer cases and 9.67 million deaths worldwide. China alone contributed 4.77 million incident cases (25.48% of the global total) and 2.56 million deaths (26.47%) [2], with malignancies now ranking as the second leading cause of mortality in urban residents (24.61%) and the third in rural populations (22.47%) [3], underscoring the urgency of cancer control.

Accelerated population aging, lifestyle changes, and environmental pollution have expanded China’s cancer patient pool, predicting further escalation of the oncologic burden [4–5]. Population aging enlarges the susceptible demographic base, lifestyle shifts introduce new and persistent carcinogenic exposures, and environmental degradation adds additional exogenous risks; these forces converge to elevate both the absolute number of cancer cases and the overall disease burden across the population. [6–7].China Health Service Survey revealed a rise in cancer-related hospitalization rates from 1.1% in 2003 to 5.8% in 2018 [8], signaling rapidly growing demand for oncology services. Against constrained resources, “difficult hospitalization” for cancer patients has become pervasive, necessitating evidence-based allocation of oncology resources.

The Chinese government has implemented policies like the Healthy China 2030 initiative and Cancer Prevention and Control Implementation Plan (2019–2022) to promote equitable health resource distribution and enhance cancer care capacity. However, vast geographic disparities and uneven economic development raise unanswered questions about spatial equity in oncology resource allocation.

At present, research on the equity of healthcare resource allocation is mostly focused on human resources, such as nursing staff [9], pharmacists [10], physicians [11–12], etc. Hospital beds, as a fundamental hardware resource, serve as a core indicator of service accessibility. Specifically, oncology bed allocation directly determines patients’ treatment access and clinical outcomes. A region’s oncology bed capacity fundamentally determines whether cancer patients can receive timely surgical intervention, chemotherapy, and radiotherapy—treatments that are largely dependent on inpatient admission. Therefore, oncology bed allocation not only directly reflects service accessibility but also serves as a key determinant of actual service utilization and quality of care.Yet, systematic analyses of oncology bed distribution remain absent in global literature.

Equitable allocation of oncology beds is fundamental to ensuring that cancer patients across different regions have fair access to necessary inpatient care, yet this critical issue has received insufficient empirical attention. This study systematically evaluates the equity status and temporal trends in oncology bed resource allocation across China from 2008 to 2022, with dual objectives: (1) to characterize spatial distribution patterns and identify disparities in oncology bed allocation, thereby assessing equity to inform policy recommendations for resource optimization; and (2) to address critical research gaps through a comprehensive longitudinal analysis of nationwide cancer care resource distribution, while advancing the theoretical framework for healthcare resource equity assessment.

## Materials and methods

### Data source

The number of oncology beds by province (autonomous region/municipality) was extracted from the China Health Statistics Yearbook (2007–2023), an authoritative annual publication released by the National Health Commission of China that compiles national and provincial-level statistics on healthcare development and population health. Oncology beds were defined as fixed, staffed beds in hospital oncology departments, with all beds in specialized cancer hospitals included.Population data were obtained from the China Statistical Yearbook (2007–2023), which is published by the National Bureau of Statistics of China and provides official national and provincial demographic data, and land area statistics came from the administrative division records of China’s Ministry of Civil Affairs.Regional classifications (eastern, central, western) followed the China Health Statistics Yearbook criteria:eastern China (11 provinces/municipalities): Beijing, Tianjin, Hebei, Liaoning, Shanghai, Jiangsu, Zhejiang, Fujian, Shandong, Guangdong, Hainan; central China (8 provinces): Shanxi, Jilin, Heilongjiang, Anhui, Jiangxi, Henan, Hubei, Hunan;western China (12 provinces/autonomous regions/municipalities): Inner Mongolia, Guangxi, Chongqing, Sichuan, Guizhou, Yunnan, Tibet, Shaanxi, Gansu, Qinghai, Ningxia, Xinjiang.All data exclude Hong Kong, Macao and Taiwan.

### Lorenz curves

The Lorenz curve is a graphical tool originally developed in economics to evaluate the equity of resource or income distribution. Currently, it is commonly used to measure the equity of healthcare resources.When applying the Lorenz curve to assess health resource equity, both population and geographic dimensions can be analyzed. For the population dimension, a coordinate system is established with the cumulative percentage of the population and the cumulative percentage of each health resource as coordinates; for the geographic dimension, the cumulative percentage of land area and the cumulative percentage of each health resource are used [13]. Specifically, in this study, the Lorenz curve ranks the number of oncology beds per 10,000 population (or per 10,000 km²) in ascending order. The x-axis represents the cumulative percentage of population (or geographic area) from 2008 to 2022, while the y-axis shows the cumulative percentage of hospital oncology beds during the same period. A line of absolute equality (diagonal) is drawn by connecting the origin and the coordinate points; this line serves as the reference for equity assessment. The closer the curve approaches this line, the higher the equity level, whereas greater curvature indicates lower equity [14].

### Gini coefficients

The Gini coefficient(G), which can more precisely reflect the equity of oncology bed resource allocation in this study, is calculated based on the Lorenz curve. Ranging from 0 to 1, values closer to 0 indicate higher equity, while values approaching 1 signify lower equity. According to established standards: a G below 0.2 represents absolute equity; 0.2–0.3 indicates relative equity; 0.3–0.4 suggests moderate equity; 0.4–0.5 reflects relative inequity; and values exceeding 0.5 demonstrate significant inequity [15–17]. The G formula employed in this study is as follows:


$$\begin{array}{c} G=\text{1}-\sum_{i=\text{1}}^{n}{(X}_{i}-X_{i-\text{1}})(\;Y_{i}+Y_{i-\text{1}}) \end{array}$$

In the formula, n indicates the number of regions, i corresponds to the i-th region after sorting healthcare resources in ascending order, X_i_ denotes the cumulative percentage of the resident population (or geographic area), Y_i_ represents the cumulative percentage of oncology bed resources.

### Theil index

The Theil index(T), ranging from 0 to 1, is a widely adopted statistical measure for evaluating resource allocation equity in academic research. Lower values indicate reduced distribution disparities and relatively higher equity. A distinctive feature of the T is its decomposability into inter-group and intra-group inequality components, which enables precise identification of the underlying causes of inequitable oncology bed allocation in China through analysis of respective contribution rates. This analytical advantage addresses the inherent limitation of the G that only measures overall inequality. The formal expression is as follows [18–20]:


$$\begin{array}{c} {\text{T}}_{intra}\text{=}\sum_{i=1}^{I}P_{i}log\frac{P_{i}}{Y_{i}} \end{array}$$

$$\begin{array}{c} {\text{T}}_{inter}\text{=}\sum_{i=1}^{I}P_{i}\sum_{i=1}^{J}P_{ij}log\frac{P_{ij}}{Y_{ij}} \end{array}$$

$$\begin{array}{c} \text{T}{\text{=T}}_{intra}{\text{+T}}_{inter} \end{array}$$

China is divided into three major regions (I = 3): the east, the central and the west. T_inter_ reflects the differences between regions. Pi is the proportion of population (geographical area) in group i in the national population (geographical area), and Yi is the proportion of oncology beds in group i in the national oncology beds; T_intra_ reflects the differences within each region, where P_ij_ represents the proportion of population (geographical area) in j province in group i, and Y_ij_ represents the proportion of oncology beds in j province in the reorganization of group i. Inter group difference contribution rate = T_inter_/T, intra group difference contribution rate = T_intra_/T.

## Results

### Allocation of oncology bed resources in Chinese hospitals

**Fig 1:**
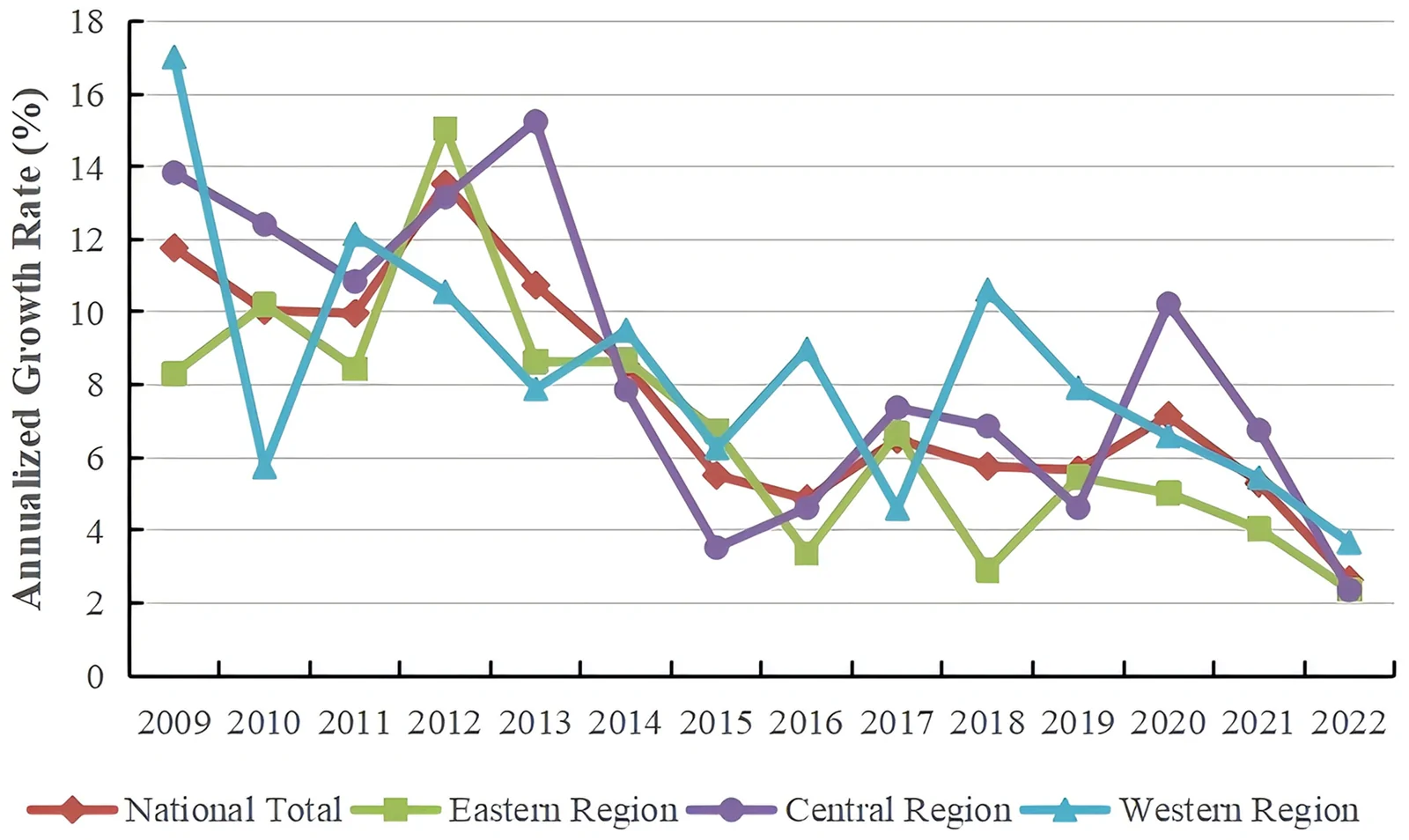
Annual growth rate of oncology beds across regions in China, 2008-2022.

From 2008 to 2022, the total number of oncology beds in Chinese hospitals increased from 99,368–279,644, representing a growth rate of 181.42%. Across the eastern, central, and western regions, all areas exhibited an upward trend in oncology bed numbers, with the central region showing the highest growth rate (212.51%), followed by the western (205.24%) and eastern (151.17%) regions.In terms of the average annual growth rate, the period from 2009 to 2013 witnessed relatively rapid expansion, peaking in 2012 (13.52%). Subsequently, the growth rate slowed, declining to 2.63% by 2022. The eastern and central regions reached their peak annual growth rates in 2012 (15.04%) and 2013 (15.24%), respectively, while the western region peaked earlier in 2009 (17.01%) (Fig 1).

**Fig 2:**
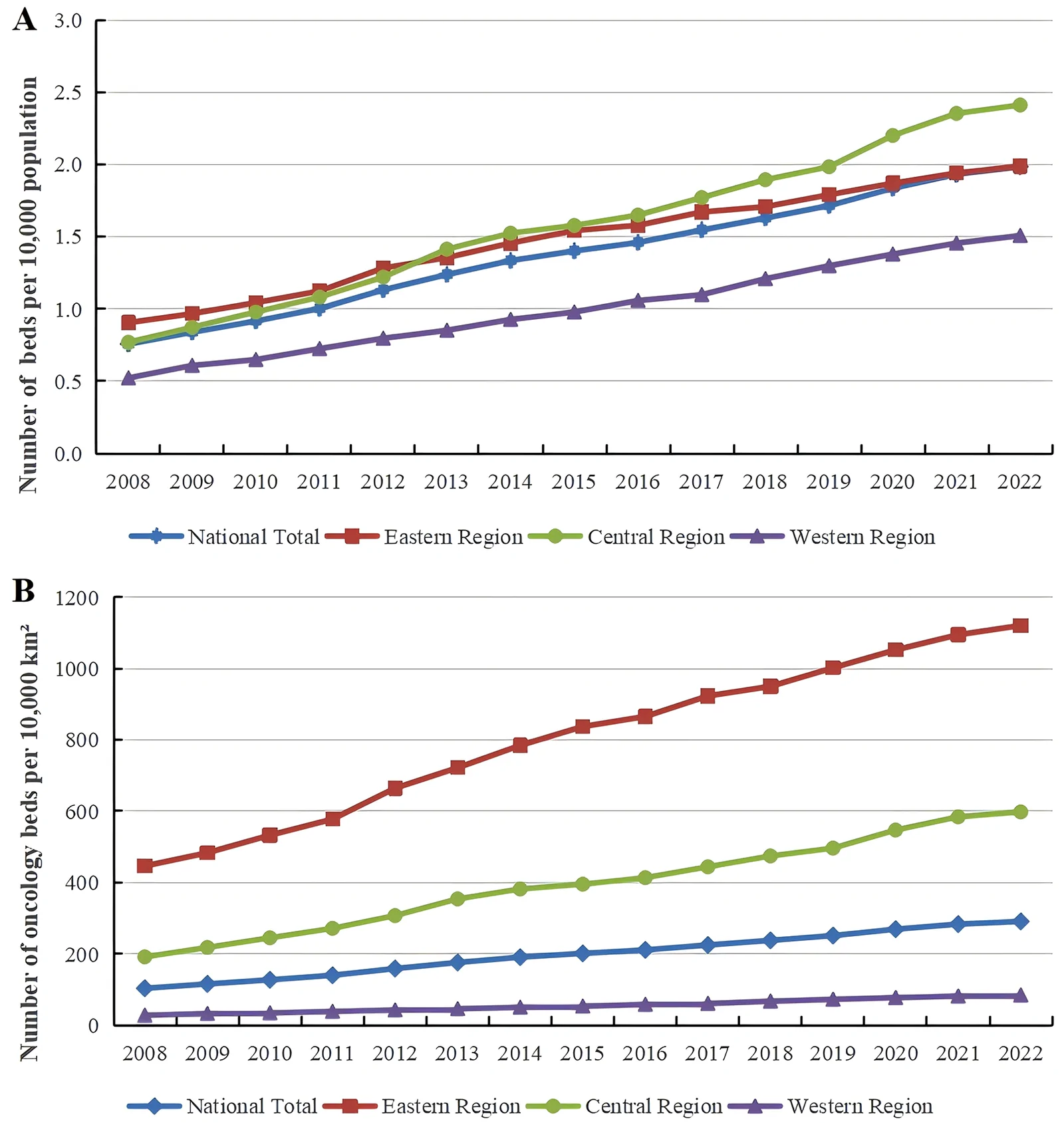
Trends in oncology bed allocation by population and geographic distribution across Chinese regions, 2008-2022.

From the perspective of population allocation, the number of hospital beds per 10,000 people in China has increased from 0.76 in 2008 to 1.98 in 2022, from 1.00 to 1.99 in the eastern region, from 0.77 to 2.41 in the central region, and from 0.52 to 1.51 in the western region. Jilin, Liaoning and Heilongjiang ranked the top three with 3.59, 3.05 and 3.05 beds per 10,000 people respectively, while Tibet, Ningxia and Yunnan ranked the bottom three with 0.12, 1.09 and 1.19 beds per 10,000 people respectively. From the perspective of geographical area allocation, the number of beds per 10,000 square kilometers in Chinese hospitals has increased from 103.30 in 2008 to 290.72 in 2022, from 446.20 to 1120.70 in the eastern region, from 191.31 to 597.85 in the central region, and from 27.62 to 84.31 in the western region. The number of beds per 10,000 square kilometers in Shanghai, Beijing and Tianjin ranked the top three, 9441.64, 4066.25 and 3202.50 respectively, while Tibet, Qinghai and Xinjiang ranked the bottom three, 0.35, 15.03 and 21.11 respectively. (Fig 2, Table 1)

**Table 1:** National oncology bed allocation by population and geographic area in 2008, 2015, and 2022.

Region	2008			2015			2022		
	Beds	Beds/10^4^ population	Beds/10^4^ km²	Beds	Beds/10^4^ population	Beds/10^4^ km²	Beds	Beds/10^4^ population	Beds/10^4^ km²
China	99368	0.76	103.30	193502	1.40	201.16	279644	1.98	290.72
eastern Region	48115	1.00	446.20	90288	1.54	837.29	120850	1.99	1120.70
central Region	32331	0.77	191.31	66781	1.58	395.15	101036	2.41	597.85
western Region	18922	0.52	27.62	36433	0.98	53.18	57758	1.51	84.31
Beijing	3325	1.88	2078.13	4570	2.09	2856.25	6506	2.98	4066.25
Tianjin	1939	1.65	1615.83	3204	2.23	2670.00	3843	2.82	3202.50
Hebei	3671	0.53	193.21	7840	1.07	412.63	11567	1.56	608.79
Shanxi	2729	0.80	170.56	5237	1.49	327.31	7688	2.21	480.50
Inner Mongolia	1831	0.75	15.52	2937	1.20	24.89	5070	2.11	42.97
Liaoning	5470	1.27	364.67	10071	2.32	671.40	12789	3.05	852.60
Jilin	2863	1.05	150.68	5585	2.14	293.95	8423	3.59	443.32
Heilongjiang	3525	0.92	76.63	5699	1.61	123.89	9459	3.05	205.63
Shanghai	3328	1.55	5249.21	4091	1.66	6452.68	5986	2.42	9441.64
Jiangsu	7456	0.96	677.82	17124	2.06	1556.73	20363	2.39	1851.18
Zhejiang	4947	0.95	494.70	9006	1.50	900.60	10666	1.62	1066.60
Anhui	4539	0.74	324.21	9828	1.64	702.00	13523	2.21	965.93
Fujian	2772	0.76	231.00	4315	1.08	359.58	5493	1.31	457.75
Jiangxi	2725	0.62	160.29	5650	1.26	332.35	9346	2.06	549.76
Shandong	7642	0.81	477.63	15657	1.59	978.56	23516	2.31	1469.75
Henan	8534	0.91	502.00	16133	1.66	949.00	23848	2.42	1402.82
Hubei	3904	0.68	205.47	10012	1.71	526.95	13247	2.27	697.21
Hunan	3512	0.55	167.24	8637	1.31	411.29	15502	2.35	738.19
Guangdong	7159	0.72	397.72	13109	1.12	728.28	18332	1.45	1018.44
Guangxi	2699	0.56	112.46	4857	1.01	202.38	7418	1.47	309.08
Hainan	406	0.48	119.41	1301	1.38	382.65	1789	1.74	526.18
Chongqing	1060	0.37	129.27	2927	0.95	356.95	6503	2.02	793.05
Sichuan	3902	0.48	79.63	9040	1.10	184.49	13914	1.66	283.96
Guizhou	973	0.27	54.06	2485	0.67	138.06	4874	1.26	270.78
Yunnan	1690	0.37	43.33	3378	0.72	86.62	5563	1.19	142.64
Tibet	37	0.13	0.31	50	0.15	0.42	42	0.12	0.35
Shaanxi	2255	0.61	107.38	3761	0.98	179.10	5362	1.36	255.33
Gansu	1351	0.53	31.42	2724	1.08	63.35	3634	1.46	84.51
Qinghai	369	0.67	5.13	592	1.03	8.22	1082	1.82	15.03
Ningxia	297	0.48	45.00	690	1.01	104.55	791	1.09	119.85
Xinjiang	2458	1.15	14.81	2992	1.25	18.02	3505	1.35	21.11

### Equity analysis of oncology bed resources in Chinese hospitals based on lorenz curve and gini coefficient

**Fig 3:**
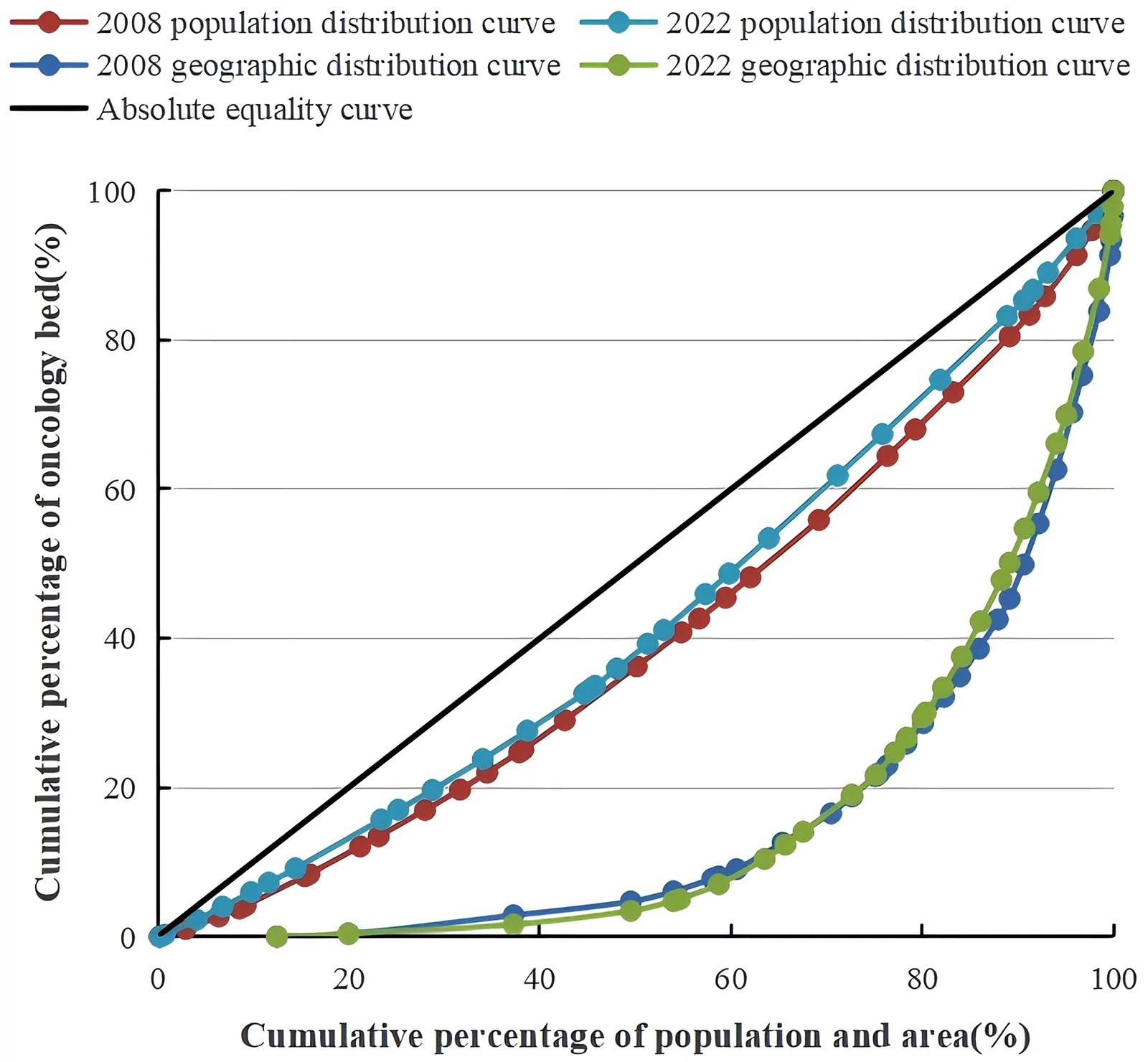
Lorenz curves for oncology bed distribution by population and geographic area in China, 2008 vs. 2022.

This article selects Lorenz curves from 2008 and 2022 for comparative analysis. The Lorenz curve distributed by population is located near the absolute equity line, while the Lorenz curve distributed by geographical area deviates more from the absolute equity line, indicating that the equity of hospital oncology bed allocation based on population is better than that based on geographical area in China. Compared to 2008, the area between the Lorenz curve based on population distribution and the absolute mean line is shrinking in 2022, and the Lorenz curve based on geographical area distribution is basically overlapping(Fig 3).

From the perspective of G, the G of hospital oncology beds based on population allocation from 2008 to 2022 is between 0.15 and 0.20, which is in an absolute equity state and shows an overall downward trend, decreasing from 0.20 in 2008 to 0.16 in 2022, indicating a significant improvement in allocation equity; The G of the number of oncology beds in hospitals based on geographical area allocation remains around 0.69, which is always in a very unfair state. (Table 2)

**Table 2:** Gini coefficients of oncology bed distribution by population and geographic area in China, 2008-2022.

Year	By population	By geographical
2008	0.20	0.69
2009	0.18	0.69
2010	0.18	0.69
2011	0.17	0.69
2012	0.17	0.70
2013	0.17	0.70
2014	0.17	0.70
2015	0.16	0.70
2016	0.16	0.69
2017	0.17	0.69
2018	0.17	0.69
2019	0.16	0.69
2020	0.15	0.68
2021	0.15	0.68
2022	0.16	0.69

### Equity analysis of oncology bed resources in Chinese hospitals based on theil index

From 2008 to 2022, the equity of hospital oncology bed resources allocation based on population is relatively good in China, with Theil indices all less than 0.1. The equity of allocation based on geographical area is relatively poor, with indices all greater than 0.6. The T based on population allocation decreased from 0.030 in 2008 to 0.019 in 2022, indicating an improvement in allocation equity. The T based on geographic area allocation increased from 0.614 in 2008 to 0.698 in 2022, indicating a decreasing degree of allocation equity. Horizontally comparing, whether in terms of population or geography, the T within each group is greater than that between groups, indicating that the unequity in allocation mainly comes from within each region. The contribution rate of intra group differences in the population dimension fluctuated from 66.04% in 2008 to 64.37% in 2022, indicating that regional differences are narrowing; The contribution rate of intra group differences in geographical dimensions has increased from 52.83% in 2008 to 62.43% in 2022, indicating that regional differences are expanding. (Table 3)

**Table 3:** Theil index decomposition of oncology bed resource allocation in China, 2008-2022.

Dimensions	Year	T	intra-group		inter-group	
			T-intra	Contribution rate (%)	T-inter	Contribution rate (%)
Population	2008	0.03	0.02	66.04	0.01	33.96
	2009	0.026	0.019	71.32	0.008	28.68
	2010	0.025	0.017	67.59	0.008	32.41
	2011	0.021	0.014	66.49	0.007	33.51
	2012	0.021	0.013	61.28	0.008	38.72
	2013	0.023	0.013	59.13	0.009	40.87
	2014	0.022	0.013	59.69	0.009	40.31
	2015	0.02	0.011	56.97	0.009	43.03
	2016	0.02	0.013	64.7	0.007	35.3
	2017	0.022	0.014	63.48	0.008	36.52
	2018	0.02	0.014	68.06	0.007	31.94
	2019	0.018	0.013	68.69	0.006	31.31
	2020	0.018	0.011	62.82	0.007	37.18
	2021	0.018	0.011	61.27	0.007	38.73
	2022	0.019	0.012	64.37	0.007	35.63
Geographical	2008	0.614	0.325	52.83	0.29	47.17
	2009	0.614	0.338	55.09	0.276	44.91
	2010	0.63	0.343	54.52	0.286	45.48
	2011	0.636	0.356	55.94	0.28	44.06
	2012	0.67	0.383	57.08	0.288	42.92
	2013	0.686	0.392	57.12	0.294	42.88
	2014	0.69	0.398	57.72	0.292	42.28
	2015	0.678	0.387	57.14	0.291	42.86
	2016	0.673	0.394	58.47	0.28	41.53
	2017	0.678	0.393	58.04	0.284	41.96
	2018	0.674	0.403	59.77	0.271	40.23
	2019	0.671	0.405	60.42	0.265	39.58
	2020	0.679	0.413	60.86	0.266	39.14
	2021	0.692	0.427	61.69	0.265	38.31
	2022	0.698	0.436	62.43	0.262	37.57

**Fig 4:**
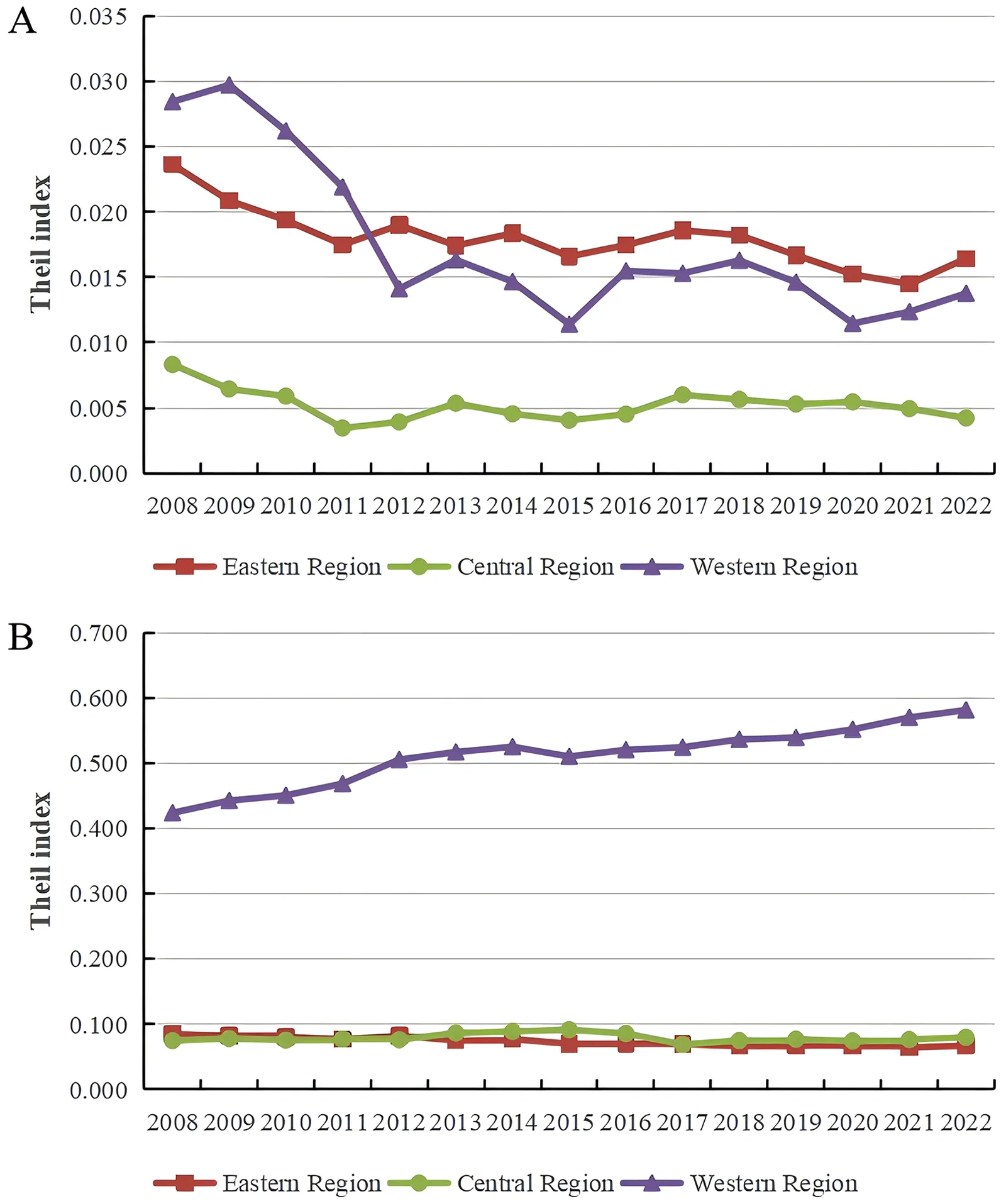
Theil indices for oncology bed resource allocation across Chinese regions, 2008-2022. Note: A is by population; B is based on geographical area.

From the population distribution of the three major regions of the east, central, and west, the T showed a downward trend from 2008 to 2022. Before 2011, the T was highest in the west, followed by the east, and lowest in the central. After 2011, the T in the west sharply decreased, and the T in the east became the highest. From the perspective of the T configured by geographical area, the western region has the highest T, while the central and eastern regions are at a lower level. (Fig 4)

## Discussion

This study presents a comprehensive analysis of the allocation patterns and equity in oncology bed resources across Chinese hospitals over a 15-year period. Our findings demonstrate significant expansion of oncology bed capacity under China’s healthcare reform policies. Specifically, per capita bed availability increased from 0.76 to 1.98 beds per 10,000 population (2008–2022), while geographic density rose from 103.30 to 290.72 beds per 10,000 km², indicating sustained resource growth.

From the perspective of equity in resource allocation, research has found that the allocation of oncology bed resources exhibits a clear binary differentiation between population and geography. From 2008 to 2022, the G of hospital oncology beds based on population allocation decreased from 0.20 in 2008 to 0.16 in 2022, maintaining a highly fair state, and the Lorenz curve shows a continuous improvement in equity. However, the G allocated by geographical area has always been greater than 0.6, which is highly unfair, and equity has not been significantly improved over the past decade. The equity of distribution based on population is much greater than that based on geographical area, which is consistent with previous research results [12,21–23].

This result confirms that China’s medical and health resource allocation has long followed the planning principle of “population based” [24], while relatively neglecting the consideration of geographical spatial factors, resulting in relatively abundant resources in densely populated areas, while sparsely populated areas face the dilemma of resource scarcity. Given that the accessibility of medical services is influenced by both population distribution and geographic space, it is recommended that a dual dimensional planning framework of “population geography” be established for future resource allocation: focusing on optimizing the equity of population dimension allocation in population agglomeration areas; To address the persistent geographic inequity identified in this study—where the Gini coefficient for geographic allocation consistently exceeded 0.6—it is necessary to establish a hierarchical diagnosis and treatment network and optimize transportation accessibility in sparsely populated areas to increase the number of oncology beds in large hospitals within the region, thereby improving the equity of health service utilization for cancer patients. In particular, the western region has only 84.31 beds per 10,000 km², far below the 1,120.70 beds per 10,000 km² in the eastern region, making transportation infrastructure improvement and the development of regional medical centers priority interventions.

The decomposition results of the T show that regional differences are the main factor affecting the equity of bed resource allocation in the oncology department of Chinese hospitals. From 2008 to 2022, the intra group contribution rate of the T configured by population dimension was 56.97% to 71.32%, while the intra group contribution rate of the T configured by geographical dimension was 52.83% to 62.43%. This indicates that the imbalance in resource allocation is mainly due to differences between provinces within the region, rather than overall differences between regions, which is consistent with relevant research results [25–28]. It is suggested that in the future allocation of hospital oncology bed resources, a differentiated regional regulation mechanism should be established to implement total quantity control for provinces with excessive resource allocation, give policy support to areas with insufficient resources, and focus on narrowing the resource allocation gap within the region. At the same time, it is possible to consider establishing regional medical cooperation bodies to promote the cross regional flow and sharing of high-quality resources, and to promote the balanced development of bed resources.

This study has several limitations. First, it mainly analyzes the equity of oncology bed resource allocation from the perspectives of population and geography, without incorporating region-specific health needs (e.g., cancer incidence and mortality) or actual service demand. Second, it focuses exclusively on oncology bed resources without examining the allocation of oncology-related human resources (e.g., oncologists, oncology nurses, and other specialized staff), which are equally critical for cancer care delivery. Third, the evaluation of resource allocation efficiency is insufficient, as this study examines bed quantity (counts and density) but does not capture bed occupancy rates or service volume, which are important dimensions of actual service capacity. Future research should integrate both material and human resource dimensions, incorporate disease burden data, and combine methods such as data envelopment analysis to achieve a more comprehensive assessment of oncology resource allocation equity that addresses both fairness and efficiency.

In summary, while China’s oncology bed resources are expanding in scale, they are facing dual challenges of imbalanced “population geography” allocation and significant regional differences. In the future, a multi-dimensional and differentiated resource allocation system should be established, with a focus on improving geographical accessibility and regional balance, effectively addressing the problem of “difficult hospitalization” for cancer patients, and providing strong support for the implementation of the Healthy China strategy.

## Abbreviations

G: Gini coefficient
T: Theil index
